# Predictors of outcome in patients with haematological malignancies admitted to critical care

**DOI:** 10.1186/cc13231

**Published:** 2014-03-17

**Authors:** A Tridente, K Browett, J Hall, Y Sorour, J Snowden, S Webber

**Affiliations:** 1 St Helens and Knowsley, Liverpool; 2 STH, Sheffield, UK; 3 DBH, Doncaster, UK

## Introduction

Critical care (CC) admission has traditionally been viewed as likely to result in a poor outcome for immune-compromised haemato-oncological (HO) patients [1,2]. Recent studies have challenged such views [3,4] We recently reported results from a cohort of HO patients admitted to CC, showing the pAO_2_/FiO_2 _(P/F) ratio to be the only independent predictor of mortality [5]. We report analyses of a larger cohort of HO patients admitted to CC.

## Methods

We assessed outcome for HO patients at CC (primary outcome) and hospital discharge, and at 6-month and 1-year follow- up. Single variable logistic regression analyses, adjusted by age, gender and haematological diagnosis, and multivariate analyses were performed to identify independent predictors of outcome using STATA.

## Results

A total of 225 HO patients were admitted to CC. Median age was 59 (interquartile range (IQR) 46 to 66) years. The most common haematological diagnoses on admission were acute myeloid leukaemia in 57 (25.3%) cases, non-Hodgkin lymphoma in 54 (24%) cases and multiple myeloma in 42 (18.7%) patients. Median APACHE II score was 21 (IQR 17 to 26). A total of 164 patients (72.9%) had at least one organ supported. Unit and hospital mortality rates were 34.7% (78 patients) and 49.3% (111 patients), respectively. At 6-month and 1-year follow-up, mortality increased to 63.1% (142 patients) and 70.5% (153 patients), respectively. The APACHE II score (OR = 0.93, 95% CI = 0.89 to 0.97, *P *< 0.001), number of organs supported (OR = 0.34, 95% CI = 0.23 to 0.48, *P *< 0.001), P/F ratio (OR = 1.06, 95% CI = 1.03 to 1.09, *P *< 0.001), inotropic requirement (OR = 0.27, 95% CI = 0.15 to 0.49, *P *< 0.001), and IMV status (Or = 0.06, 95% CI = 0.03 to 0.14, *P *< 0.001) influenced unit survival at single variable analyses. At multivariate analysis, the P/F ratio (OR = 1.05, 95% CI = 1.02 to 1.09, *P *= 0.002) and IMV status (OR = 0.12, 95% CI = 0.04 to 0.35, *P *< 0.001) independently predicted outcome.

## Conclusion

Organ failures and need for organ support correlated with outcome. P/F ratio and need for IMV were independent predictors of mortality, in agreement with previously published data [6]. The CC mortality rate was 34.7%; at 1 year, mortality had risen to 70.5%.
